# Theoretical Studies Applied to the Evaluation of the DFPase Bioremediation Potential against Chemical Warfare Agents Intoxication

**DOI:** 10.3390/ijms19041257

**Published:** 2018-04-23

**Authors:** Flávia V. Soares, Alexandre A. de Castro, Ander F. Pereira, Daniel H. S. Leal, Daiana T. Mancini, Ondrej Krejcar, Teodorico C. Ramalho, Elaine F. F. da Cunha, Kamil Kuca

**Affiliations:** 1 Laboratory of Molecular Modeling, Chemistry Department, Federal University of Lavras, 37200-000 Lavras, MG, Brazil; flaviavillela09@yahoo.com.br (F.V.S.); alexandre.a.castro@hotmail.com (A.A.d.C.); ander.francisco@hotmail.com (A.F.P.); daniel.leal@ufes.br (D.H.S.L.); daianateixeira60@yahoo.com.br (D.T.M.); teo@dqi.ufla.br (T.C.R.); elaine_cunha@dqi.ufla.br (E.F.F.d.C.); 2 Department of Health Sciences, Federal University of Espírito Santo, 29932-540 São Mateus, ES, Brazil; 3 Center for Basic and Applied Research, Faculty of Informatics and Management, University Hradec Kralove, 50003 Hradec Kralove, Czech Republic; ondrej.krejcar@uhk.cz

**Keywords:** organophosphorus compounds, DFPase, tabun, cyclosarin, soman, molecular docking, PCA, QM/MM

## Abstract

Organophosphorus compounds (OP) are part of a group of compounds that may be hazardous to health. They are called neurotoxic agents because of their action on the nervous system, inhibiting the acetylcholinesterase (AChE) enzyme and resulting in a cholinergic crisis. Their high toxicity and rapid action lead to irreversible damage to the nervous system, drawing attention to developing new treatment methods. The diisopropyl fluorophosphatase (DFPase) enzyme has been considered as a potent biocatalyst for the hydrolysis of toxic OP and has potential for bioremediation of this kind of intoxication. In order to investigate the degradation process of the nerve agents Tabun, Cyclosarin and Soman through the wild-type DFPase, and taking into account their stereochemistry, theoretical studies were carried out. The intermolecular interaction energy and other parameters obtained from the molecular docking calculations were used to construct a data matrix, which were posteriorly treated by statistical analyzes of chemometrics, using the PCA (Principal Components Analysis) multivariate analysis. The analyzed parameters seem to be quite important for the reaction mechanisms simulation (QM/MM). Our findings showed that the wild-type DFPase enzyme is stereoselective in hydrolysis, showing promising results for the catalytic degradation of the neurotoxic agents under study, with the degradation mechanism performed through two proposed pathways.

## 1. Introduction

The use of chemical substances in wars was boosted in the 1930s with the discovery of the toxic properties of organophosphorus compounds (OP). Since then, the interest in the synthesis of substances that present toxicity intensified, and more potent compounds were developed, such as the neurotoxic agents, which later came to be used as chemical weapons [1].

**Figure 1:**
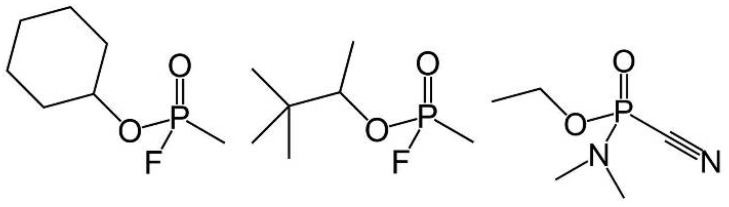
Structures of the neurotoxic agents Cyclosarin (**left**), Soman (**center**) and Tabun (**right**), respectively.

These OP agents present structural characteristics which cause them have different activities, being employed as insecticides, herbicides, plant growth regulators, therapeutic agents and chemical weapons [2,3,4]. The members of this family, called nerve agents or neurotoxic agents, are the most lethal [5], due to their high toxicity. Given these characteristics, they have potential for use as chemical warfare agents, being considered as mass destruction weapons [2]. They are classified as G- or V-type agents, being Tabun (GA), Sarin (GB), Soman (GD), Cyclosarin (GF) and VX the main representatives of this class [1]. Some of these chemical weapons are represented in Figure 1.

The G-type neurotoxic compounds have one chiral center, but Soman presents two stereocenters. It is noteworthy that one stereoisomer (*S_P_*) is commonly more toxic than the other (*R_P_*) [6]. Concerning Soman, biological essays related to acetylcholinesterase (AChE) inhibition processes are only capable of discriminating the stereoisomers pair taking into account the phosphorus atom, which is the dominant chiral center that dictates the potential toxicity, being this stereocenter generally employed to theoretical investigations [7].

The intoxication process through neurotoxic agents takes place in the central and peripheral nervous system [8], where these compounds inhibit the AChE enzyme. As a consequence of this inhibition, there may be the collapse of the nervous system, and finally death [9,10].

**Figure 2:**
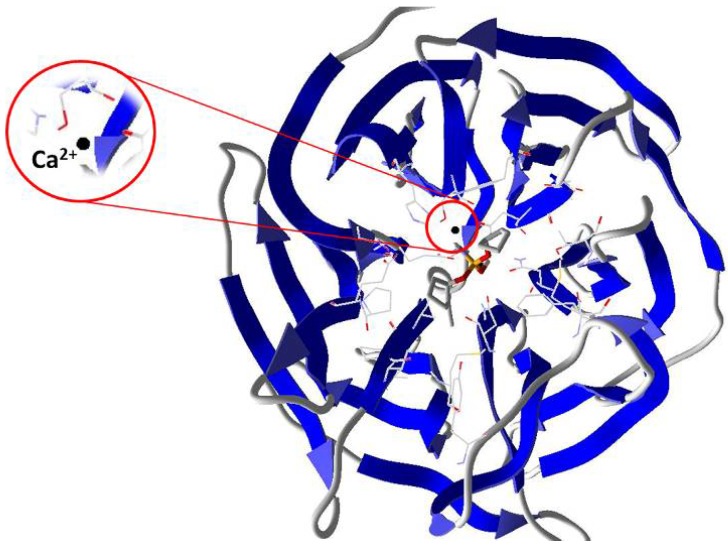
Representation of DFPase crystallographic structure (PDB code 2GVV) [13]. The DFPase structure is shown as solid ribbons. The original ligand (DcPPA) is shown as tubular bonds.

In this context, the employment of enzymes capable of degrading OP shows up as an alternative treatment [11]. Among the enzymes investigated with this applicability, the Diisopropyl fluorophosphatase (DFPase), from the squid *Loligo vulgaris*, has emerged recently, showing a good potential for this purpose [12]. DFPase is a Ca^2+^-dependent phosphotriesterase, which has been investigated as a potent biocatalyst for the hydrolysis of a series of highly toxic OP [7]. The DFPase crystallographic structure with its original bonded ligand (dicyclopentylphosphoroamidate–DcPPA) is shown in Figure 2.

**Figure 3:**
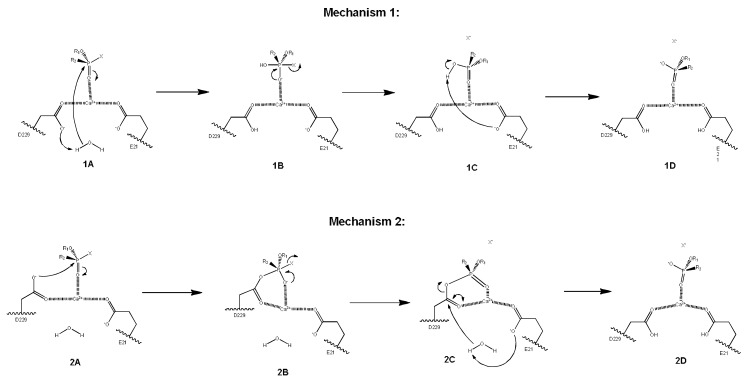
OP hydrolysis reaction pathways proposed for DFPase action. Mechanism 1: it is shown the participation of a molecule of water as nucleophile with assistance of both Asp229 (D229) and Glu21 (E21) residues. In 1A, the hydroxide ion formed with the assistance of Asp229 attacks the OP leading to the pentavalent intermediate. In 1B, this intermediate collapses, hence, producing the leaving group X^−^. In 1C, the Glu21 residue removes a proton of the OP, leading to 1D. Mechanism 2: only the Asp229 residue participates as nucleophile on the reaction. In 2A, Asp229 attacks the OP. In 2B, the pentavalent intermediate collapses. In 2C, a molecule of water hydrolyses the intermediate with the assistance of Glu21, leading to 2D, which is the same structure as 1D. In Mechanism 2, the tetrahedric intermediate was not shown for clarity reasons. In both mechanisms, the X^−^ leaving group is stabilized by other residues (not shown). Adapted from references [7,11,12,13].

Previous studies have suggest an essential role of the Asp229 residue in the DFPase catalytic activity, since it has the correct orientation to perform a nucleophilic attack in the hydrolysis mechanism through bimolecular nucleophilic substitution (S_N_2), and it is also coordinated to the Ca^2+^ ion together with other residues and water molecules [7,11,12,13]. One possible pathway for the catalysis is that the Asp229 acts as a nucleophile, attacking the OP coordinated to the Ca^2+^ ion. In another reaction pathway for DFPase, in which the OP substrate is again coordinated to the Ca^2+^ catalytic ion via phosphoryl oxygen, a water molecule is activated through Asp229 (by proton abstraction) and then the hydroxyl ion formed acts as a nucleophile on the phosphoric center. The Glu21 participates in these proton transfers. The leaving group is released and the hydrolyzed substrate is removed from the Ca^2+^ ion at the ionized form as the reaction product [7,11,12,13]. Simplified versions of both these proposed mechanisms are represented in Figure 3.

Although there are previous studies [7,11,12,13] that assess the potential degradation of OP through DFPase, there is yet no consensus in the literature describing the exact reaction pathway. This feature indicates the need for a better understanding of the hydrolysis mechanisms and reactivity for the presented bioremediation process. Based on the existing proposals, the present work suggests a more complete evaluation, which evaluates the stereochemistry of these compounds, considering the stereoselectivity of the enzyme in the hydrolysis.

Given that the story of use of OP as chemical weapons is old, recent reports have made the scenario worrying and indicate that the development, production, and use of OP are a major reason to give due attention to the issue, dealing with it as a real threat present to the whole world. Although there are certain resources available for treatment, there is not a universal antidote efficient against all existing OP agents. In this context, considering that the bioremediation is an expanding field, and that the knowledge about the wild-type DFPase enzyme has brought significant contributions for the medicinal chemistry, more studies become necessary [10,14,15]. Thus, this work uses theoretical molecular docking studies, supported by chemometric analyzes, along with computations such as quantum mechanics/molecular mechanics (QM/MM) to investigate the OP degradation through DFPase enzyme.

## 2. Results and Discussion

### 2.1. Multivariate Exploratory Analysis

Once the molecular docking is performed, the MVD^®^ software reports provided poses or conformers in the enzyme active site, with their respective interaction energy values. Usually, the selection of the conformation (pose) for the reaction mechanism studies is based on the lower energy criterion. However, considering the complexity of the system in which the mechanism takes place, it is believed that there are other relevant parameters, which may also assist in the choice of this conformation [16]. From this, a data matrix, composed of 30 conformations and 8 variables, was constructed and the multivariate exploratory analysis was used to try to detect association patterns of this data set.

The fundamental basis of most methods for the treatment of multivariate data is the Principal Component Analysis (PCA), which was employed in this study. In this kind of analysis, the original data set, correlated or not, are decomposed into a set of uncorrelated orthogonal variables, called principal components (PC). Then, the new set is described in a coordinate system which is expressed in terms of the linear combination of the initial variables, and it is possible to identify the individual contribution of each variable [17].

The new variables are obtained in decreasing order of the statistical information amount that they describe, i.e., the first principal component (PC1) points the direction of the largest variation of the data; the second one (PC2), which is orthogonal to the first one, points another direction that describes the largest remaining variation of the data, and so on.

The technique allows the reduction of the dimensionality of the representative points of the samples, even if the statistical information present in the n original variables is the same as in the n principal components, being common to obtain in only 2 or 3 of the first principal components about 70% to 80% of this information. Based on this, it can be seen in Table 1 that more than 70% of the data variation was explained in the first three principal components, for all compounds. In other hands, the presented data indicate that this new set of values, represented by PC1, PC2 and PC3, is more informative, since it can stand for the same system in a smaller number of dimensions.

The most common way to graphically represent the results of the PCA decomposition is to plot, on a graph, the samples and weights of the principal components chosen, most often creating a two-dimensional graph that allows a clearer view of the arrangement of these samples (graphs PC1 versus PC2 projection) and the contribution of the initial variables (loadings graph of the variables investigated). In this context, the analysis of principal components in the loadings graphs can also be used to judge the importance of the original variables selected.

**Figure 4:**
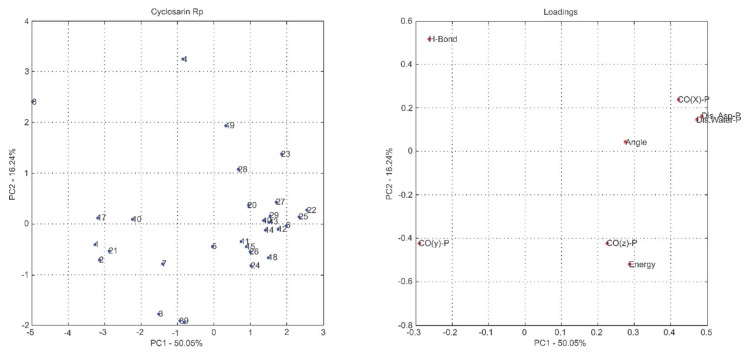
PC1 versus PC2 projection for Cyclosarin (*R_P_* enantiomer) and loadings graph of the variables investigated (**left** and **right**, respectively).

**Figure 5:**
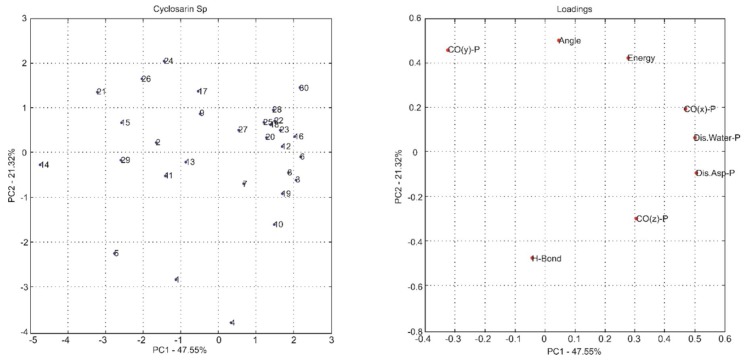
PC1 versus PC2 projection for Cyclosarin (*S_P_* enantiomer) and loadings graph of the variables investigated (**left** and **right**, respectively).

**Figure 6:**
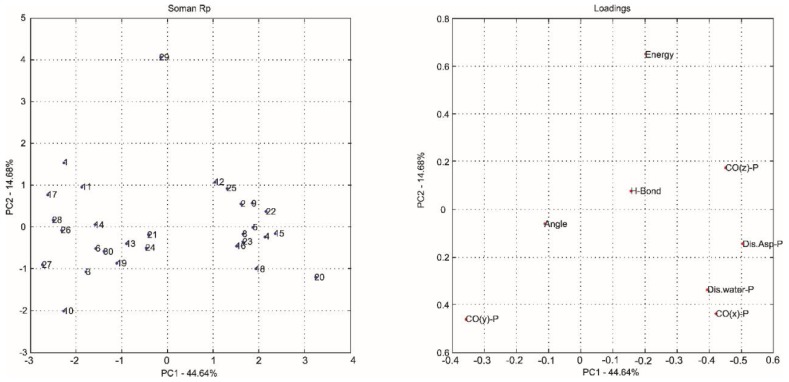
PC1 versus PC2 projection for Soman (*R_P_* enantiomer) and loadings graph of the variables investigated (**left** and **right**, respectively).

**Figure 7:**
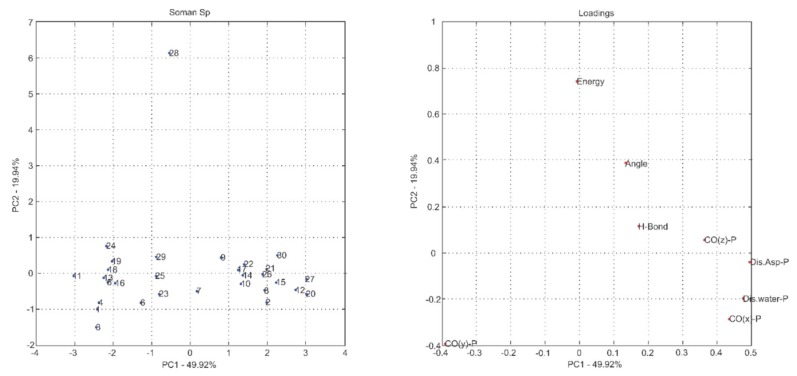
PC1 versus PC2 projection for Soman (*S_P_* enantiomer) and loadings graph of the variables investigated (**left** and **right**, respectively).

**Figure 8:**
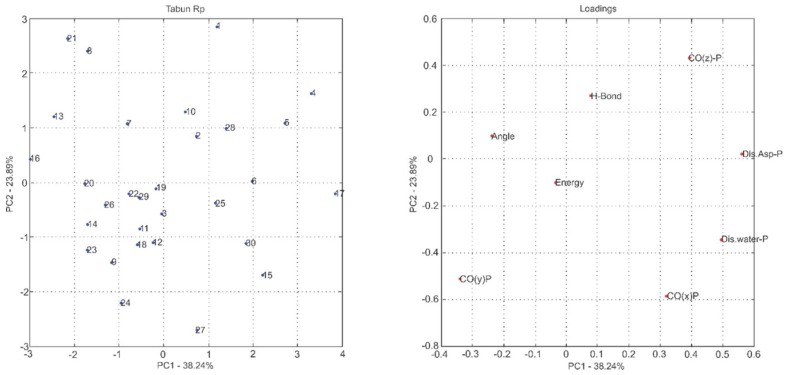
PC1 versus PC2 projection for Tabun (*R_P_* enantiomer) and loadings graph of the variables investigated (**left** and **right**, respectively).

**Figure 9:**
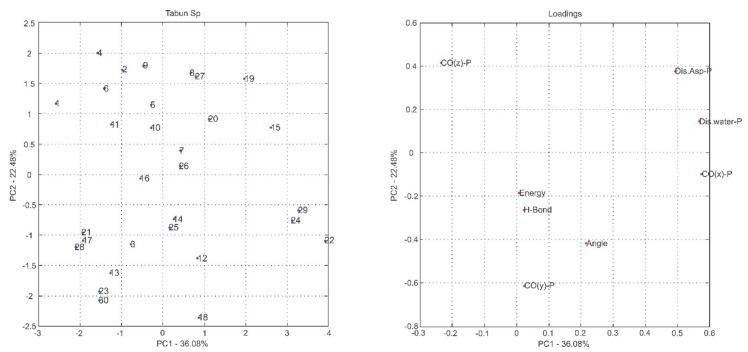
PC1 versus PC2 projection for Tabun (*S_P_* enantiomer) and loadings graph of the variables investigated (**left** and **right**, respectively).

Considering that the interpretation of the loading graphs indicates the most important original variables for the linear combination of PC, from the statistical point of view, Figure 4, Figure 5, Figure 6, Figure 7, Figure 8 and Figure 9 reveal which parameters or variables may be considered. In all cases, the common variables for both enantiomers were judged. Considering Cyclosarin, the variables obtained in the positive region of the graph are angle, Co(X)P and Dist H_2_O-P. For Tabun, only Dist Asp-P was observed in the region established for this analysis. On the other hand, for Soman, along with H-bonds and Co(z)P, the energy also includes the variables of larger weight. Thus, in addition to energy, other parameters, such as the spatial position of the phosphorus atom, deserve to be taken into account for the selection of the pose that stands for the OP in the hydrolysis reaction mechanism.

Considering that the energy itself cannot be the only parameter used to choose the conformation that best represents the OP in the reaction mechanism, the PCA method was employed as an alternative for this selection [18]. The selection method used is based on the idea of occupancy rate and has the objective of choosing the pose that contributes less to the standard deviation of the median structure. For this, bidimensional representations were built to maximize the interpretation of the data and to allow the indication of a set of poses suitable for the mechanism simulation.

It is known in PCA that the explanation percentage of PC1 will always be higher than that of PC2, and so on. Thus, the projections that may represent the data sets are: PC1 vs. PC2 or PC1 vs. PC3, since PC_N_ > PC_N+1_. The PC1 vs. PC2 plan has a greater explanation of the original data set, so the proposal here was to use it [18]. Thus, the projections from Figure 4, Figure 5, Figure 6, Figure 7, Figure 8 and Figure 9 report the dispersion of the poses as a function of the first and second PCs.

The selection is performed based on the pose that is closest to the origin point (0, 0) of the PC1 versus PC2 projection. Arbitrarily, a boundary was delimited (−1, +1) to facilitate the indication of the structure. The poses 5, 13, 21, 7, 19 and 26, generated on docking calculations, were indicated by PCA to stand for the neurotoxic agents (*R_P_*)-Cyclosarin, (*S_P_*)-Cyclosarin, (*R_P_*)-Soman, (*S_P_*)-Soman, (*R_P_*)-Tabun and (*S_P_*)-Tabun, respectively, in the reaction mechanism simulation. Theoretically, they are the most representative of the original data set, presenting a higher similarity to the median structure.

### 2.2. Affinity: Molecular Docking

Molecular docking calculations were performed to adjust the ligands (OP) in the enzyme active cavity, evaluating the affinity among them. For this study, a cavity prediction algorithm based on a 3D box was employed in order to generate the DFPase enzyme binding sites. The volume of the calculated active cavity was 164.864 Å^3^.

Some parameters were collected and investigated in order to understand the interaction modes of the OP in the study within the wild-type DFPase active site and to investigate the factors which contribute to the degradation of these compounds. Table 2 shows the intermolecular interaction energy values and the hydrogen bonds performed by the nerve agents, with amino acid residues and water molecules present in the active site.

**Figure 10:**
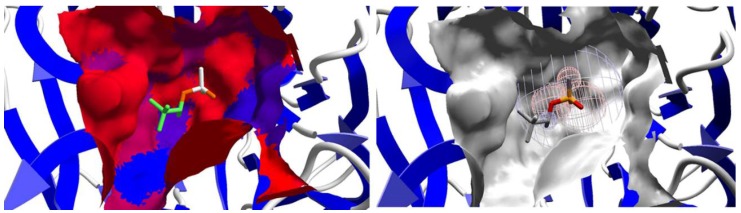
Representation of the hydrophobic (on the left) and electrostatic (on the right) interactions of Soman (*R_P_* enantiomer) in the DFPase active cavity, wherein red surface means hydrophobic regions and blue surface means hydrophilic regions.

It is possible to notice, according to Table 2, that the nerve agent (*R_P_*)-Soman owns the lowest intermolecular interaction energy value (−40.88 kcal·mol^−1^). However, no hydrogen bond was detected for this compound, which indicates that there are other factors that contribute to its stabilization in the enzyme active site. Probably, the conformation adopted by this compound is favored by hydrophobic and electrostatic interactions, as observed in Figure 10.

**Figure 11:**
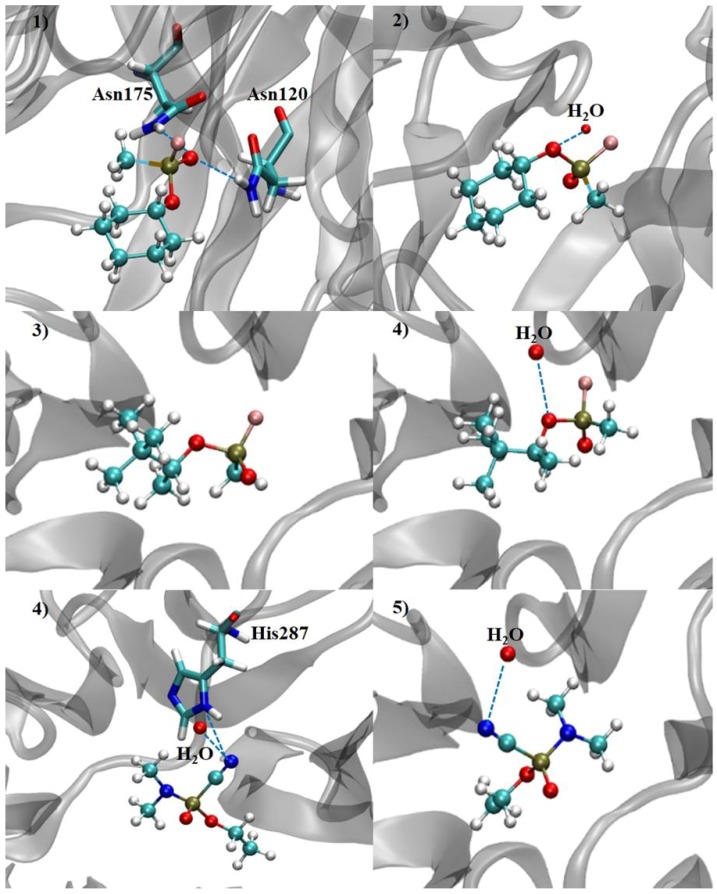
Hydrogen bonds performed by the nerve agents in the DFPase active site (Cyclosarin: *R_P_* (**1**) and *S_P_* (**2**) enantiomers; Soman: *R_P_* (**3**) and *S_P_* (**4**) stereoisomers; Tabun: *R_P_* (**5**) and *S_P_* (**6**) enantiomers).

On the other hand, (*R_P_*)-Cyclosarin, which presented the second lowest interaction energy value (−39.23 kcal·mol^−1^), carried out hydrogen bonds with the Asn120 and Asn175 amino acid residues, which brought about contributions for the stabilization of the compound in the active site (Figure 11). Similar to the above-mentioned compound, (*R_P_*)-Tabun has a good interaction potential with DFPase, since interactions with the His287 amino acid residue was observed, in addition to a water molecule (Figure 11). It is important to keep in mind that the *R_P_* enantiomers of Cyclosarin and Tabun allowed a better interaction and stabilization in the active site, and this conformation led to the formation of hydrogen bonds with amino acid residues, unlike the *S_P_* enantiomer.

The *S_P_* enantiomers of both OP agents performed only one hydrogen bond with one water molecule (Figure 11), and presented the highest interaction energy values, which were −32.34 kcal·mol^−1^, −28.87 kcal·mol^−1^ and −28.33 kcal·mol^−1^ for Cyclosarin, Tabun and Soman, respectively. These results point out that, as observed for other degrading enzymes, the DFPase active site demonstrates a stereochemical preference for one enantiomer in relation to the other. However, this fact can be evaluated by means of the theoretical reaction mechanism simulation. 

### 2.3. Mechanistic Studies in the Wild-Type DFPase Active Site

The development of new strategies, aiming to design new and selective organophosphorus degrading agents is important. For this purpose, the understanding of the acting means of these compounds becomes necessary [16]. In this context, the QM/MM approach can be used in order to have a good understanding of the interaction and reactional modes between OP and DFPase [14]. For this purpose, we also performed a theoretical investigation of the wild-type DFPase performance, considering the reaction mechanism of hydrolysis. The results obtained for the activation energy, based on the proposed mechanisms (Figure 3) can be observed in Table 3.

**Figure 12:**
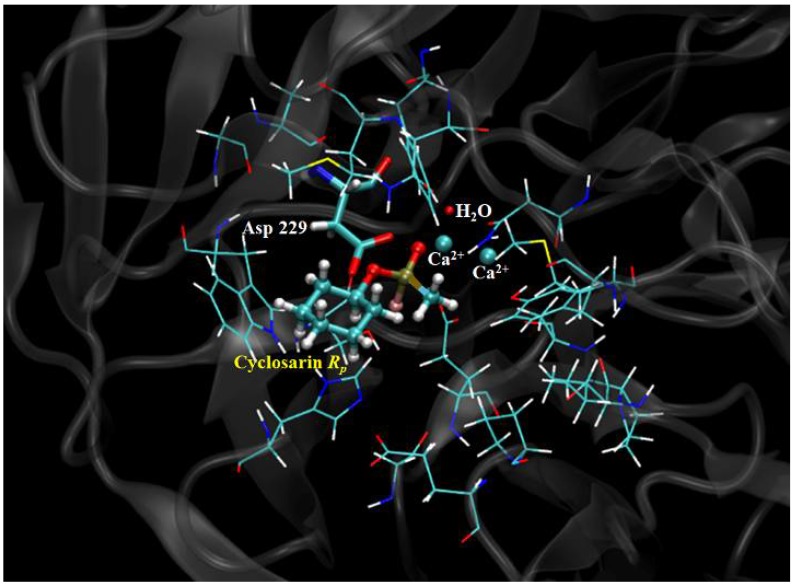
QM reactional system (highlighted in coloured atoms) for the Cyclosarin (*R_P_* enantiomer) degradation by DFPase, considering a radius of 5 Å. All coloured residues were taken into account for the QM reactional system.

In order to perform this theoretical procedure, the hydrolysis reaction mechanism was looked into by employing two distinct routes, as shown in Figure 3. According to the QM system displayed in Figure 12, the metal ion is responsible for the catalytic activity of the enzyme, and it has a key role in this reaction, given that these cofactors are crucial for the water molecule activation step, giving rise to a hydroxyl ion which directly attacks the phosphoric center of the neurotoxic agent. It is important to keep in mind that the stereochemistry dictates the neurotoxicity level of these chemical weapons, influencing a lot in the degradation process, because it is found that one enantiomer could be preferentially hydrolyzed in relation to the other. These data can be observed in Table 3.

The experimental studies performed by Chen et al. [6] exhibit the importance and potential of DFPase for application in the bioremediation of the poisoning caused by neurotoxic OP agents. Their experimental results suggest how the DFPase behaves in the hydrolysis of Cyclosarin, taking into account the *R_P_* and *S_P_* enantiomers. The experimental values of catalytic efficiency (*K*_cat_/K_m_) were presented, and according to these results, the *R_P_* enantiomer of Cyclosarin is more efficiently degraded, with a catalytic efficiency value equal to 7.2 × 10^5^ M^−1^ s^−1^. On the other hand, DFPase showed a lower efficiency in the hydrolysis of the *S_P_* enantiomer (*K*_cat_/K_m_ = 1.7 × 10^4^ M^−1^ s^−1^) [6]. This tendency is, according to our theoretical results, obtained from the reaction mechanism simulation. A deeper study was performed theoretically, and significant aspects that govern the DFPase action on these agents have been figured out by means of diverse computational methodologies, allowing us to have a better comprehension of this enzyme in the bioremediation process.

By observing Table 3, it is possible to notice that the enzyme has shown a big selectivity regarding the Cyclosarin degradation. In this case, the *R_P_* enantiomer revealed a lower activation energy value in relation to the *S_P_* enantiomer, with an energy difference of 9.79 kcal·mol^−1^ (via Mechanism 1), thus being more efficiently degraded. The same tendency can be noticed via Mechanism 2, wherein the energy difference was about 30.34 kcal·mol^−1^, corroborating very well with the experimental values of catalytic efficiency. The highest selectivity is found from the Mechanism 2 pathway; this must be strongly related to the fact that the hydrolysis process begins with the attack of a bulkier species, which is the carboxylate portion of Asp229 in this case. Therefore, this species acts as the nucleophile by this route, undergoing a higher steric hindrance in the degradation of Cyclosarin (*S_P_* enantiomer). Furthermore, the selectivity was not so remarkable compared to Mechanism 1, because in this case, the hydroxyl ion formed in the water activation step, which acts as a nucleophile in the S_N_2 mechanism, is less bulky, not suffering significant steric hindrance. As can be seen in Figure 1, the nerve agent Cyclosarin presents one cyclohexane ring in its structure, further enhancing the steric hindrance on the nucleophile.

**Figure 13:**
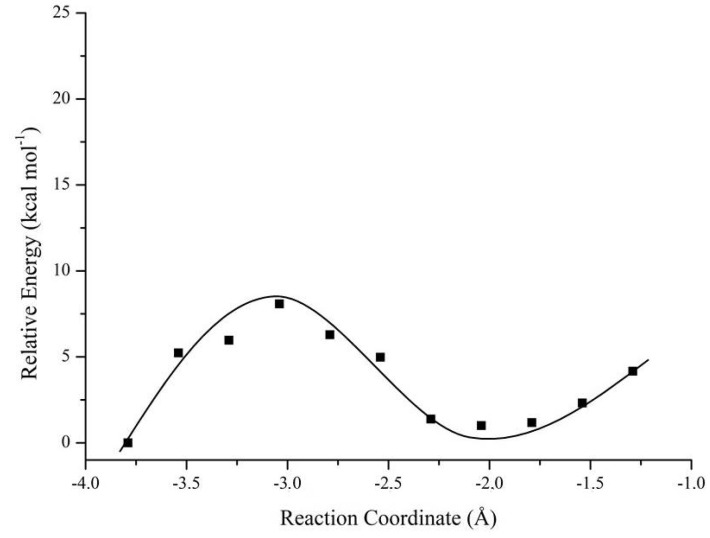
Graph of relative potential energy versus reaction coordinate for Soman (*S_P_* enantiomer).

Now evaluating the DFPase performance in the Tabun degradation, we can notice that the same tendency continues. The *R_P_* enantiomer is more efficiently degraded in relation to *S_P_* enantiomer, via both mechanism routes. Another important fact to be mentioned here is that the selectivity was not so apparent, no matter the pathway. The energy differences were very small, being 0.16 kcal·mol^−1^ (via Mechanism 1) and 2.42 kcal·mol^−1^ (via Mechanism 2). The selectivity from Mechanism 2 is slightly higher, maybe due to the steric hindrance effects shown right behind, but for Tabun, the presence of very bulky substituents in its structure is not observed, unlike Cyclosarin. Finally, we evaluated the degradation process of the neurotoxic agent Soman, and things have changed in relation to the preferential stereochemistry in the hydrolysis. For both mechanism routes, the *S_P_* enantiomer of Soman was preferentially degraded, with an energy difference of 9.98 kcal·mol^−1^ (via Mechanism 1) and 3.72 kcal·mol^−1^ (via Mechanism 2). The graph of potential energy versus reaction coordinate is highlighted in Figure 13, for Soman (*S_P_* enantiomer), which presented a relative energy barrier equal to zero. It is quite interesting that Soman, even presenting a bulky group, did not interfere most in the process, and the transition state via Mechanism 2 is found to be more stable, i.e., the reaction intermediates are better stabilized by following this hydrolysis pathway. However, both mechanisms investigated here are likely to take place and, sometimes, this choice will depend on the compound structure (substituents attached to the central phosphorus), its stereochemistry and leaving group in the S_N_2 mechanism.

Further experimental studies were developed by Dawson et al. [16], and they investigated the factors involved in the Soman degradation, and some experimental value was displayed. For the Soman degradation (*R_P_* enantiomer), we found a value of catalytic efficiency of 1.44 × 10^5^ M^−1^ s^−1^. According to this experimental data [16], along with the experimental results from Chen et al. [6], it is noteworthy the good correlation between theoretical and experimental results. According to them, the nerve agent Cyclosarin owns the highest catalytic efficiency involving hydrolysis, in relation to Soman, and this tendency is also seen by means of the theoretical calculations performed, wherein Cyclosarin presents the lowest activation energy for the process, with an energy difference of about 3.85 kcal.mol^−1^. These experimental and theoretical studies are a good starting point to have a better comprehension of the acting modes of degrading enzymes (wild-type DFPase for example), thus, allowing the development of new technologies which are cheap and efficient in the remediation of the intoxication caused by these neurotoxic agents [19].

Concerning Soman, it is worth noting that, although the research was carried out considering the phosphorus atom as the predominant stereocenter, the extension of the theoretical studies directed to the two stereoisomers (*S*,*R_P_*) and (*R*,*S_P_*), regarding the carbon chiral, must be considered. This aspect deserves further investigations in order to support the validation of the model. In fact, with this promising lead in hand, we next sought to improve the understanding of this reaction. Further and still more accurate theoretical calculations in order to verify this hypothesis are now in progress. 

## 3. Materials and Methods

### 3.1. Computational Details

#### 3.1.1. Docking Procedure

For the docking study, the nerve agents Tabun, Soman and Cyclosarin were used to perform the calculations. Chemical structures of both *R_P_* and *S_P_* stereoisomers of these compounds (with the subscript index corresponding to the phosphorus atom as the stereocenter) were constructed and individually optimized by using the Gaussian 09 package [20], at DFT level, with B3LYP density functional and 6-31G(d,p) basis set, wherein the partial charges of the atoms were elucidated. Just in the case of Soman, which has four stereoisomers, it was used only the variations (*R*,*R_P_*) and (*S*,*S_P_*), given that the different configurations on the phosphorus atom are responsible for the OP toxicity. For this work, considering that the chiral carbon is irrelevant to determine the toxicity of this neurotoxic agent, an equal pair of stereoisomers considering both chiral centers (*R*,*R_P_*) and (*S*,*S_P_*) was thus chosen [7]. The *R_P_* and *S_P_* stereoisomers of these compounds were individually docked inside the crystallographic structure of DFPase (PDB code 2GVV; resolution = 1.73 Å) [13], using the Molegro Virtual Docker program (MVD^®^) [21], taking into account the same procedures employed in other studies and the previous removal of the original ligand DcPPA [22,23,24]. For the development of the docking calculations, it was considered a radius of 5 Å centered at the active site, with the residues being kept as flexible. Due to the nature of docking methods, the calculations were executed generating 100 poses (conformation and orientation) for each stereoisomer of the ligands.

The MolDock scoring function employed in the MVD^®^ program comes from the piecewise linear potential (PLP), a simplified potential whose parameters are fitted to protein-ligand structures, binding data scoring functions and further extended in Generic Evolutionary Method for molecular docking with a new hydrogen bonding term and new charge schemes [25]. The docking scoring function values, E_score_, are defined by Equation (1):

(1) $$E_{\mathrm{score}}{\;=\;E}_{\mathrm{inter}}{\;+\;E}_{\mathrm{intra}}$$

where:

(2) $$E_{\mathrm{inter}}\;=\underset{\;i\varepsilon \mathrm{ligand}}{\sum }\underset{j\varepsilon \mathrm{protein}}{\sum }\left[E_{\mathrm{PLP}}\left(r_{\mathrm{ij}}\right)\;+\;332.0\frac{\mathrm{qiqj}}{{4r}_{\mathrm{ij}}^{2}}\right]$$

E_PLP_ represents “piecewise linear potential”, which consists in the use of two different parameter sets, as described forward: one for approximation of the steric term (Van der Waals) among atoms, and the other potential for the hydrogen bonding. The second term is related to the electrostatic interactions among overloaded atoms. It is a Coulomb potential with a dielectric constant dependent on the distance (D_(r)_ = 4r). The numerical value of 332.0 is responsible for the electrostatic energy unit to be given in kilocalories per molecule [21].

E_intra_ is the internal energy of the ligand:

(3) $$E_{\mathrm{intra}}\;=\;\underset{i\varepsilon \mathrm{ligand}}{\sum }\underset{j\varepsilon \mathrm{ligand}}{\sum }E_{\mathrm{PLP}}\left(r_{\mathrm{ij}}\right)\;+\;\underset{\mathrm{flexiblebonds}}{\sum }A\left[1-\cos\left(m{.\theta -\theta }_{0}\right)\right]{\;+\;E}_{\mathrm{clash}}$$

The first part of the equation (double summation) is among all pairs of atoms in the ligand, taking off those which are connected by two bonds. The second one characterizes the torsional energy, where θ is the torsional angle of the bond. If several torsions could be determined, each torsional energy is considered and there is the use of an average among them. The last term, E_clash_, assigns a penalty of 1000 if the distance between two heavy atoms (more than two bonds apart) is smaller than 2.0 Å, not taking into account infeasible ligand conformations [21]. The docking search algorithm that is applied in MVD^®^ program considers an evolutionary algorithm, the interactive optimization techniques which are inspired by Darwinian evolution theory, and a new hybrid search algorithm called guided differential evolution. This hybrid combines the differential evolution optimization technique with a cavity prediction algorithm during the search process, allowing that way a fast and accurate identification of potential binding modes (poses) [21,26,27].

#### 3.1.2. Multivariate Analysis of Principal Components

Taking into account the complexity of the analyzed system, and the large amount of data obtained, the multivariate analysis was carried out by employing relevant information from the molecular docking, in order to look into the proposed reaction mechanism.

Initially, starting from the 100 conformations generated by the docking calculations, 30 low energy poses were selected. The data matrix was constructed as pre-processing, extracting from the docking an amount of 8 variables considered important for the mechanism simulation. This generated matrix is composed of lines, which represent the samples (OP compounds), and columns representing the original variables to be investigated. These are: (x, y, z) space coordinates for the phosphorus atom of the nerve agents ((CO(x)-P), (CO(y)-P), (CO(z)-P)), intermolecular interaction energy (ENERGY), hydrogen bonds (H-BOND), attack angle to the OP (ANGLE), distance between the oxygen of theAsp229 amino acid residue and phosphorus in the OP (DIS.ASP-P) and distance between the water molecule and P in the OP (DIS.WATER-P).

The method is based on the transformation of original variables (present in the data matrix) into new uncorrelated variables, called principal components (PC). The main components are obtained by means of linear transformations according to the equation:

X × P = T(4)

wherein X is the original matrix of the data, T is the scores matrix containing the coordinates of the samples in the new axis system and P is the loadings matrix, where the elements of each column correspond to the coefficients of the linear combinations of the original variables.

For this statistical treatment, the MATLAB program, version R2012b, was used [17]. We have considered the closest water molecule and the Asp229 amino acid residue, which presented a favorable spatial location for the reaction mechanism. The data matrix underwent a pretreatment process by employing self-escalation. 

#### 3.1.3. QM/MM Methodology

Combined QM/MM techniques allow the modeling of larger systems, like reactions within enzymes, by combining the electronic degrees of a quantum chemical approach with the MM methods, increasing the performance and decreasing computational demand [10]. Thus, in this work, mixed quantum and molecular mechanics (QM/MM) combined with docking techniques were performed to determine the most likely reaction pathway for the OP degradation catalyzed by the wild-type DFPase. Actually, this theoretical strategy has been previously employed in other occasions [25,28,29,30], being intensively used in our current researches, and so, computational methods are necessary for allowing us to analyze them [10]. In an attempt to get more accurate results and electronic effects, QM calculations were carried out at the density functional theory (DFT) level with the Gaussian 09 package [20]. DFT methods have been widespread, showing good performance for large systems, such as biomolecules [31,32]. The calculations were based on the generalized gradient approximation functional proposed by Gustin et al. [33]. This relationship of functional and basis sets has been tested for similar systems [26].

All transition states, intermediates and precursors involved were calculated and characterized by calculations identifying imaginary frequencies [5,34,35]. Each conformer was fully optimized at DFT level, with conjugate gradient and quasi-Newton-Raphson algorithms. The final geometries were obtained with the density functional Becke’s three-parameter exchange functional and the gradient-corrected functional of Lee, Yang and Paar (B3LYP) [36], using 6-31G(d,p) basis set. The QM system consisted in neighboring peptide bonds, link atoms, crystallographic water molecules, Asp229, GA/GD/GF (*R_P_* and *S_P_* stereoisomers), inside a sphere within a radius of 5 Å. Within this boundary, it was possible to include the electrostatic and hydrophobic effects from diverse residues, which assist in the stabilization and reaction modes over the reaction pathway.

Given the significant importance of electrostatic interactions for the ligands stabilization in the enzyme active site, all residues important electrostatically were part of the QM reactional system, concerning to the reaction mechanism simulation, being these residues found within a radius of 5 Å, as mentioned before. By employing quantum calculations, it was possible to include these electronic effects in the simulation, which are essential for a better stabilization of the transition state. 

Some important features regarding the QM/MM approach are as follows: a small part of the system is selected for QM treatment, for instance, by carrying out the calculations at ab initio or semi-empirical level, or DFT, as in the present work [37,38]. The quantum mechanics (QM) approach is a method which potentially allows the electronic rearrangements referred to the breaking and formation of chemical bonds. In this theoretical study, the QM region consisted of the enzyme active site, as described previously. The big portion of the system, not directly related to the reaction pathway, is treated by empirical molecular mechanics (MM) techniques [38]. In the performance of this kind of calculations, the QM/MM energy of the whole system, E_TOTAL_^QM/MM^, is calculated as shown in Equation (1):

(5) $$E_{\mathrm{TOTAL}}^{\mathrm{QM}/\mathrm{MM}}{\;=\;E}_{\mathrm{TOTAL}}^{\mathrm{MM}}{\;+\;E}_{\mathrm{QM}\;\mathrm{region}}^{\mathrm{QM}}{\;-\;E}_{\mathrm{QM}\;\mathrm{region}}^{\mathrm{MM}}$$

where $E_{\mathrm{TOTAL}}^{\mathrm{MM}}$ is the MM energy of the whole system, $E_{\mathrm{QM}\;\mathrm{region}}^{\mathrm{QM}}$ is the QM energy of the QM region and $E_{\mathrm{QM}\;\mathrm{region}}^{\mathrm{MM}}$ is the MM energy of the isolated QM region. Many QM/MM techniques still include the polarization effect on the QM region by the MM environment [39]. In this line, the QM/MM has shown itself as being a nice tool to perform these calculations.

## 4. Conclusions

Considering that currently the use of chemical warfare agents is one of the biggest threats to the world population, the advent of effective detoxification techniques of these compounds becomes essential. In this perspective, the theoretical methodology proposed in this work is highlighted, since it was able to evaluate the affinity and reactivity of these compounds in the wild-type DFPase active site, obtaining detailed data and perspectives on the central processes of the enzymatic catalysis process. Our results suggest that the enzyme owns stereochemical preference in the degradation of these compounds.

The results point to DFPase as a considerable biological tool, considering that the two pathways suggested for the degradation mechanism seem to be promising, depending on the OP structure and its stereochemistry. The agreement between the experimental results found in the literature and the theoretical ones obtained in this work characterizes the adequacy of the applied methodologies. Thus, the enzymatic catalysis predicted by the computational chemistry methods for the OP degradation serves as a starting point in order to contribute to important advances in medicinal chemistry.

## Figures and Tables

**Table 1:** Percentage of variance of the data explained by PCA for the OP and their enantiomers.

Principal Components	% of Variance	% of Cumulative VARIANCE	Principal Components	% of Variance	% of Cumulative VARIANCE
(*R_P_*)-*Cyclosarin*			(*S_P_*)-*Cyclosarin*		
PC1	50.05	50.05	PC1	47.55	47.55
PC2	16.24	66.29	PC2	21.32	68.87
PC3	14.82	81.11	PC3	12.88	81.75
PC4	10.23	91.34	PC4	8.98	90.73
PC5	5.75	97.09	PC5	5.98	96.71
PC6	2.81	99.90	PC6	3.21	99.92
PC7	0.09	99.99	PC7	0.07	99.99
PC8	0.01	100.00	PC8	0.01	100.00
(*R_P_*)-*Soman*			(*S_P_*)-*Soman*		
PC1	44.64	44.64	PC1	49.92	49.92
PC2	14.68	59.32	PC2	19.94	69.87
PC3	13.48	72.80	PC3	12.13	82.00
PC4	11.63	84.43	PC4	10.64	92.64
PC5	6.83	91.26	PC5	6.07	98.71
PC6	5.25	96.51	PC6	1.25	99.96
PC7	3.43	99.94	PC7	0.03	99.99
PC8	0.06	100.00	PC8	0.01	100.00
(*R_P_*)-*Tabun*			(*S_P_*)-*Tabun*		
PC1	38.25	38.25	PC1	36.08	36.08
PC2	23.89	62.14	PC2	22.48	58.56
PC3	16.22	78.36	PC3	15.66	74.22
PC4	12.95	91.30	PC4	11.39	85.61
PC5	5.90	97.20	PC5	8.83	94.44
PC6	2.60	99.80	PC6	5.34	99.78
PC7	0.19	99.99	PC7	0.13	99.91
PC8	0.01	100.00	PC8	0.09	100.00

**Table 2:** Values of the parameters obtained by molecular docking calculations for the OP in the DFPase active site, by employing the conformations selected in the PCA analysis (energies in kcal·mol^−1^; lengths in angstroms; units correspond to those used by default in the MVD^®^ software).

Neurotoxic Agent	Intermolecular Interaction Energy	H-Bond Strength	H-Bond Length	Residues and H_2_O	Neurotoxic Agent	Intermolecular Interaction Energy	H-Bond Strength	H-Bond Length	Residues and H_2_O
(*R_P_*)-Cyclosarin	−39.23	−1.58	2.99	Asn120	(*S_P_*)-Cyclosarin	−32.34	−2.50	2.97	H_2_O
		−2.24	2.88	Asn175					
(*R_P_*)-Soman	−40.88	-	-	-	(*S_P_*)-Soman	−28.33	−2.49	3.10	H_2_O
(*R_P_*)-Tabun	−32.80	−2.50	3.09	H_2_O	(*S_P_*)-Tabun	−28.87	−2.50	3.10	H_2_O
		−0.14	3.49	His287					

**Table 3:** Relative activation energy (ΔΔE^#^) in the hydrolysis mechanism of different nerve agents by the wild-type DFPase (units were kept in kcal.mol^−1^ for ease of comparison with docking results).

Nerve Agent	Enantiomers	Mechanism 1; ΔΔE^#^ (kcal·mol^−1^)	Mechanism 2; ΔΔE^#^ (kcal·mol^−1^)
*Soman*	*R_P_*	9.98	5.53
	*S_P_*	0.00	1.81
*Tabun*	*R_P_*	2.05	0.10
	*S_P_*	2.21	2.52
*Cyclosarin*	*R_P_*	2.11	1.68
	*S_P_*	11.90	32.02

## Abbreviations

ACh	Acetylcholine
AChE	Acetylcholinesterase
Asn	Asparagine
Asp	Aspartate
B3LYP	DFT hybrid functional by Becke, Lee, Yang and Paar
DcPPA	Dicyclopentylphosphoroamidate
DFPase	Diisopropyl fluorophosphatase enzyme
DFT	Density Functional Theory
GA	NATO designation code for tabun
GB	NATO designation code for sarin
GD	NATO designation code for soman
GF	NATO designation code for cyclosarin
His	Histidine
*K*_cat_	Turnover number
K_m_	Michaelis constant
M	Mols per liter
MM	Molecular Mechanics
MVD	Molegro Virtual Docker^®^ software
OP	Organophosphorus
PC	Principal Component
PCA	Principal Components Analysis
PLP	Piecewise Linear Potential
QM	Quantum Mechanics
*R_P_*	*R*-stereochemistry designation for the OP phosphorus atom
*S_P_*	*S*-stereochemistry designation for the OP phosphorus atom
